# The Effect of Rate-Controlling Medication on the Performance and Outcome of Dobutamine Stress Echocardiography in the Assessment of Patients with Suspected Angina: A Retrospective Cohort Study

**DOI:** 10.3390/jcm15082850

**Published:** 2026-04-09

**Authors:** Laya Hariharan, Muhammad Zohaib Amjad, Emil Tom John, Valentina Cospite, Sudipta Chattopadhyay, Attila Kardos

**Affiliations:** 1 Translational Cardiovascular Research Group, Department of Cardiology, Milton Keynes University Hospital NHS Foundation Trust, 8H Standing Way, Eaglestone, Milton Keynes MK6 5LD, UK; laya.hariharan@mkuh.nhs.uk (L.H.); muhammad.amjad@mkuh.nhs.uk (M.Z.A.); emil.john@mkuh.nhs.uk (E.T.J.); sudipta.chattopadhyay@nhs.net (S.C.); 2 Department of Cardiology, County Durham and Darlington NHS Foundation Trust, Durham DH1 5TW, UK; v.cospite1@nhs.net; 3 Department of Cardiology, Bedford Hospital NHS Trust, Bedford MK42 9DJ, UK; 4 Faculty of Medicine and Health Sciences, University of Buckingham, Buckingham MK18 1EG, UK

**Keywords:** stress echocardiography, rate controlling medical treatment, outcome, MACE

## Abstract

**Background/Objectives**: Stress echocardiography (SE) had been recommended by professional societies for assessing patients with suspected angina. SE protocols are variable across hospitals and countries in the recommendation of the cessation of rate-controlling medication (RCMx) prior to SE. Some expert opinion papers recommend the cessation of beta receptor blockers (BBs) and rate-controlling calcium channel blockers 48 h prior to SE to improve the diagnostic accuracy of the test. There is no evidence that the continuation of RCMx can affect the outcome of SE and short-term major adverse cardiovascular events (MACEs). To investigate the efficacy of Dobutamine SE in a cohort of patients where the cessation of rate-controlling medication has not been mandated, we reviewed our data over a one-year period in patients investigated for suspected coronary artery disease (CAD). **Methods**: A retrospective data analysis was performed on 227 consecutive patients who underwent Dobutamine SE between January 2022 and January 2023 in a single centre. In addition to dobutamine, the protocol allowed the administration of intravenous atropine (maximum dose of 1.2 mg) and a “top up” handgrip exercise at the discretion of the performing cardiologist. We assessed the Dobutamine SE outcome (positive vs. negative), target heart rate (THR, 85% of maximum age predicted), and the achieved peak HR in the two groups with RCMx and without RCMx. We analysed the patients’ characteristics and 12-month outcomes of a combined MACE of death, non-fatal MI, stroke, admission with angina, and unplanned revascularisation. **Results**: Of the 227 patients, 61% were on No-RCMx (male 40%). Ninety-three percent of the patients on RCMx were on BB and 7% on other rate-controlling medications. The THR was achieved in 74% of the patients with-RCMx and 90% in the without-RCMx groups *p* = 0.0018. Positive Dobutamine SE was observed in 48% (43/89) of patients on RCMx vs. 28% (39/138) on No-RCMx (*p* = 0.0022). Patients who did not reach THR 43% (16/37) had positive Dobutamine SE compared to 35% (66/190) who reached THR (*p* = 0.626). There was no difference between groups in the peak WMSI. Logistic regression analysis showed that being on RCMx was independently associated with positive Dobutamine SE (OR 2.03, 95% CI 1.06–3.91, and *p* = 0.034). The MACE rate was higher in patients where the THR was not achieved (9/37, 24.0%) vs. where THR was achieved (9/190, 4.7%), *p* < 0.001, in both the with-RCMx (7/30, 23% vs. 6/66, 9.1%, *p* = 0.013) and without-RCMx (2/14, 14% vs. 3/124, 2.4%; *p* = 0.025) groups, respectively. RCMx was independently associated with MACE (OR 3.68, 95% CI 1.227–11.046, and *p* = 0.020). **Conclusions**: The use of RCMx proved to be a predictor of both SE and MACE outcomes irrespective of the achieved THR. Our data supports the practice that patients referred for Dobutamine SE on RCMx can continue taking them without impact on the test accuracy.

## 1. Introduction

Stress echocardiography (SE) is one of the non-invasive modalities of choice, as recommended by international and national guidelines, to assess patients with suspected coronary artery disease with a pretest probability of 15–85% [1,2]. With the advent of ultrasound technology including the incorporation of the AI algorithm to enhance image quality and the broader availability of ultrasound-enhanced contrast agents to improve endocardial border detection, the diagnostic accuracy of SE has significantly increased. SE can not only safely diagnose flow-limiting coronary stenosis but, with the assessment of Doppler-based CFVR, it can also assist in the diagnosis of coronary microvascular dysfunction [1]. Beyond its utility to diagnose coronary artery disease, SE is a powerful prognostic tool to predict short-term and long-term mortality and major adverse cardiovascular events (MACEs) [3,4], as supported by the ESC and AHA guidelines at IA recommendation.

One of the termination criteria for SE is to achieve the age-predicted target heart rate (THR). Patients who did not achieve THR during SE (aka chronotropic incompetence) have been associated with poor outcomes [5,6].

Although not supported by evidence, the current SE practice, especially using exercise or dobutamine stress tests, inconsistently recommends withholding rate-controlling medications 48 h prior to the test (mainly beta receptor blockers). Withholding beta blockers may facilitate achievement of the THR, but it may put patients at risk who are stable on their antianginal medication by provoking a rebound effect and could elicit unstable symptoms, even myocardial infarction, hypertension, or arrythmia [7,8,9]. In addition, this practice adds an extra layer of complexity to schedule patients for their test and can cause anxiety to patients who were stable on their beta blockers. In addition, this patient instruction, by taking the flexibility away from the booking process, can inadvertently increase waiting times. In this retrospective study, we assessed the effect of rate-controlling medical treatment (RCMx) in prospective patients undergoing Dobutamine SE on diagnostic accuracy and short-term prognosis.

## 2. Methods

### 2.1. Patients and Protocol

In this retrospective cohort study, 227 consecutive patients were enrolled who underwent Dobutamine SE between January 2022 and January 2023 in a single centre for suspected or known coronary artery disease (CAD). Patients referred for other indications for Dobutamine SE were excluded (such as structural heart disease, viability-only assessment). Patients received patient information leaflets via a post and a QR link to a patient information video to accustom themselves with the procedure at least a week prior the test. Patients received a detailed explanation about the risks and benefits and possible side effects of the SE by the performing consultant cardiologist prior to providing their written consent on the day of the test. Patients were allowed to continue to take all their medication before SE. Patients were cannulated of their brachial vein, and a three-way tap was connected for administering dobutamine (atropine if required) and ultrasound-enhancing agents (UEA, Sonovue, Bracco, Milan, Italy or Luminity, Lantheus MedicalImaging Inc., Billerica, MA, USA) via a rotator pump (Bracco) with a set infusion rate and with bolus injection setup.

Vital signs including heart rate (HR), oxygen saturation, and monitor ECG were continuously recorded throughout the test, and systolic and diastolic blood pressure (SBP, DBP) were recorded on a 3 min basis. For patients with inducible regional wall motion (RWM) abnormality (positive SE results) and/or who developed sustained arrythmia, short-acting beta blockers (Esmolol) were given intravenously. Patients were observed for 30 min in the recovery phase before discharge.

Transthoracic echocardiograph (Philips EPIQ CVxi, X5-1 matrix transducer, Philips Medical Systems UK Limited, Surrey, UK) was used to assess the RWM of the 17 left ventricular segments at rest and during SE in five views: apical 4, 2, and 3 chamber views and the parasternal long- and short-axis views at the papillary muscle level as per clinical recommendation [10,11]. The RWM was recorded at each stage of the Dobutamine SE protocol (at rest, low-dose dobutamine, at peak/end of stress, and in recovery). Quad images were recorded for offline analysis and to generate the wall motion score index for each stage.

Dobutamine intravenous infusion in 4 stages (3 min each) started at 10 mcg/kg/min until 10 mcg/kg/min increments every three minutes to the maximum of 40 mcg/kg/min was administered. In addition to dobutamine, the protocol allowed for the administration of intravenous atropine from the beginning of stage 3 (150 mcg bolus injection to the maximum dose of 1200 mcg) and the “top up” handgrip exercise at the discretion of the performing cardiologist.

### 2.2. Outcome Measures

The primary outcome measures for this audit were twofold. Firstly, patients were stratified into two groups according to their use of RCMx, specifically beta blockers or rate-controlling calcium channel blockers. The groups were categorised as **with RCMx** (patients on RCMx) and **without RCMx** (patients not on RCMx). For each group, the THR was defined as 85% of the maximum age predicted, 0.85x[220-age]), and the achieved peak HRs during Dobutamine SE were recorded.

Secondly, the diagnostic outcome of Dobutamine SE was classified as positive or negative based on the presence of at least one new RWMA (hypokinesis, akinesis, and dyskinesis) or no RWMA induced during the stress phase, respectively.

Additional outcome measures included a comprehensive assessment of short-term prognosis by tracking the composite endpoint of major adverse cardiac events (MACEs) over a 12-month period following Dobutamine SE. MACE was defined as the occurrence of death, non-fatal myocardial infarction, stroke, hospital admission with angina, or unplanned coronary revascularisation confirmed by the study team tasked to retrieve information from the hospital’s electronic health record that is linked to the local primary care database.

We also analysed the patients’ clinical characteristics, anthropometric data, and stress echocardiography parameters. This study was initiated by the Cardiology department as a service improvement project, and it was duly registered with and approved by the Clinical Governance and Audit Committee, as well as the Institutional Research Board (reference number AUD-226). The research was conducted in accordance with the principles outlined in the Declaration of Helsinki and Good Clinical Practice guidelines.

### 2.3. Statistical Analysis

Continuous variables are presented as means with 95% confidence intervals (CIs) if data were parametric and as medians with 95% CI for non-parametric data. Categorical variables are presented as counts and percentages.

For the comparison of categorical data, the Chi square test was used. Non-parametric paired and independent sample comparisons were performed using the Wilcoxon rank test and the Mann–Whitney test, respectively. Logistic regression and stepwise regression analysis was applied to identify predictors for key outcomes, including Dobutamine SE results (positive or negative), achievement of THR (yes or no), and stepwise regression analysis to define the predictors of MACEs. The regression models were adjusted for potential confounding variables: age, sex, diabetes, obesity, dyslipidaemia, family history of premature coronary artery disease (CAD), resting and peak HR, resting and peak SBP and DBP, and the use of RCMx.

All statistical computations were carried out using MedCalc Statistical Software (version 17.0.4, Ostend, Belgium). A two-tailed *p* value of less than 0.05 was considered statistically significant.

### 2.4. Missing Data Handling

In this study, missing values were noted for several parameters: SBP and DBP at rest were absent in 13 patients, resting HR in 8 patients, and SBP and DBP at peak stress in 16 patients. Consequently, the calculated double product (SBP × HR at peak), which relies on these values, was also missing for 16 patients. To address this, we used imputation by substituting the median values for the missing data points.

Sensitivity analyses were conducted to assess the impact of missing data (Supplementary Table S1). This suggests that the imputation approach did not influence the main findings.

## 3. Results

### 3.1. General Results

Over the 12-month period, 227 consecutive patients underwent Dobutamine SE in our institution. The median age (25–75%CI) was 69 (59–76) years, and 49% were male. The detailed anthropometric variables, cardiovascular risk factor profiles, and ongoing medical treatments are listed in Table 1. It should be noted that 89 (40%) patients were on rate-controlling medication (37% on beta receptor blockers and 3% on rate-controlling calcium channel blockers). The resting HR was 71 (63–80) bpm, and the peak HR was 135 (127–144) bpm. The SBP and DBP response to Dobutamine SE is in keeping with the inodilator properties of dobutamine. Eighty-four percent of patients achieved their THR, which was 88% (85–92%). Eighty-two (36%) patients had inducible wall motion abnormality predominantly between 1 and 4 segments out of the 17 segments of the LV wall.

### 3.2. Difference in Cardiovascular Risk and Medication Between Groups of Patients with and Without RCMx (Table 1)

Of the 227 patients, 89 (39%) were with RCMx (60% male), *p* < 0.05, and had a higher prevalence of diabetes mellitus. The other anthropometric variables and cardiovascular risk factors were not statistically significant compared with patients with RCMx. Ninety-three percent of patients with RCMx were on beta receptor blockers and 7% were on rate-controlling calcium channel blockers. Patients with RCMx were more likely taking ACEi/ARB, anti-platelets, and other antianginal medications compared to the group without RCMx, *p* < 0.05.

### 3.3. The Effect of RCMx on Dobutamine SE Parameters (Table 1)

Patients with RCMx had significantly lower resting and maximum HRs (68 vs. 74 bpm and 132 vs. 140 bpm, respectively) and had lower resting DBP (72 vs. 76 mmHg) compared to patients without RCMx. Seventy-four percent of patients with RCMx achieved THR vs. 90% of patients without RCMx (*p* = 0.0018). Those who achieved their THR in both groups were slightly but significantly lower in the with-RCMx group (87% vs. 89%, *p* = 0.038). Interestingly, the double product during stress test was not statistically different between the groups (18,161 vs. 19,040 mmHg*bpm). Patients with RCMx had a higher prevalence of inducible ischaemia on Dobutamine SE (48% vs. 28%, *p* = 0.0022), with a higher number of ischaemic LV segments also supported by the WMSI peak, Delta WMSI (Table 1). Interestingly, of the patients who did not reach THR, 43% (16/37) had positive Dobutamine SE compared to 35% (66/190) who reached THR (*p* = 0.626).

### 3.4. The Effect of RCMx on Composite MACEs (Table 1 and Table 2)

All participants were retrospectively assessed for clinical outcomes over the 12-month period. There was no loss to follow-up, and those with no events were censored at the end of the 12 months following their Dobutamine SE.

**Table 2:** Subgroup characteristics as a function of achieved THR in the two groups with or without RCMx.

	WITH RCMx (N = 89)			WITHOUT RCMx (N = 138)		
	THR Achieved	THR Not Achieved	*p*	THR Achieved	THR not Achieved	*p*
N	66 (74%)	23 (26%)	<0.001	124 (90%)	14 (10%)	<0.001
Age	71 (61–77)	67 (60–73)	0.1118	69 (56–750	59 (51–66)	0.0515
Male sex	40 (61)	13 (57)	0.7325	51 (41)	7 (50)	0.5254
Height (cm)	166 (160–174)	168 (160–181)	0.2202	166 (160–173)	170 (157–177)	0.5064
Weight (kg)	84 (73–97)	79 (65–94)	0.2747	77 (67–87)	85 (80–111)	0.0127
BSA (m^2^)	1.90 (1.80–2.10)	1.90 (1.72–2.18)	0.7775	1.90 (1.70–2.00)	2.00 (1.87–2.12)	0.0131
**Cardiovascular risk factors**						
Hypertension	46 (70)	14 (61)	0.4393	69 (56)	9 (64)	0.538
Dyslipidaemia	38 (58)	11 (48)	0.4209	64 (52)	4 (29)	0.1034
Smoker	11 (17)	5 (22)	0.2154	27 (22)	1 (7)	0.3924
Diabetes mellitus	26 (39%)	11 (48%)	0.4823	27 (22)	2 (14)	0.5160
Obesity	32 (48.5%)	6 (26%)	0.0629	37 (30)	6 (43)	0.3205
Family history of CAD	13 (20)	4 (17)	0.8097	35 (28)	1 (7)	0.0897
**Medications**						
Beta receptor blockers	62 (94)	21 (91)	0.666	0 (0)	0 (0)	1.000
Rate-controlling calcium channel blockers	4 (6)	2 (9)	0.666	0 (0)	0 (0)	1.000
DHP-calcium channel blockers	22 (33)	6 (26)	0.5612	29 (23)	6 (43)	0.1138
ACE-I/ARB	38 (58)	14 (61)	0.7837	40 (32)	6 (43)	0.4269
Anti-platelets	44 (67)	16 (70)	0.799	50 (40)	6 (43)	0.8553
Nitrate	28 (42)	7 (30)	0.3135	26 (21)	3 (21)	0.9681
**DSE parameters**						
HR at rest (bpm)—imputed	69.5 (60.0–78.0)	63.0 (56.0–71.0)	0.051	73 (64–80)	72 (63–82)	0.5462
HR at peak (bpm)	135 (128–143)	116 (114–124)	<0.001	140 (130–148)	131 (126–136)	0.0139
SBP rest (mmHg)—imputed	137 (117–160)	138 (130.3–155.3)	0.305	138 (120–157)	150 (124–175)	0.2318
SBP peak (mmHg)—imputed	136 (122–153)	139 (124–169)	0.3484	136 (119–154)	135 (121–177)	0.5701
DBP rest (mmHg)—imputed	72 (64–77)	74 (67–78)	0.686	74 (68–82)	78 (72–82)	0.1965
DBP peak (mmHg)—imputed	67 (58–72)	67 (59–76)	0.502	67 (60–74)	68 (58–78)	0.7110
THR at peak (% of MTHR)	89 (87–92)	79 (71–81)	<0.001	89 (87–94)	84 (81–84)	<0.001
Double product (mmHg*bpm)	18,617 (16,320–21,708)	16,100 (14,707–18,697)	0.0189	19,040 (16,479–22,024)	18,176 (16,214–3010)	0.8105
WMSI at rest	1.00 (1.00–1.06)	1.00 (1.00–1.04)	0.7714	1.00 (1.00–1.00)	1.00 (1.00–1.06)	0.2229
WMSI at peak	1.06 (1.00–1.18)	1.06 (1.00–1.23)	0.7896	1.00 (1.00–1.06)	1.00 (1.00–1.06)	0.7208
Delta WMSI	0.00 (0.00–0.18)	0.00 (0.00–0.18)	0.7222	0.00 (0.00–0.060)	0.00 (0.00–0.060)	0.4998
Positive DSE (inducible ischaemia)	32 (48)	11 (48)	0.9568	34 (27)	5 (36)	0.5150
Biphasic response (viable ischaemic)	16 (24)	5 (22)	0.8858	14 (11)	4 (29)	0.089
**Number of LV segments with inducible ischaemia**						
0	33(50)	12 (52)	0.680	90 (73)	9 (64)	0.839
1–2	20 (30)	7 (30)	0.791	30 (24.0)	3 (21)	0.486
3–4	12 (18)	3 (13)	0.478	3 (2)	2 (14)	0.045
≥5	1 (2)	1 (4)	0.553	1 (1)	0 (0.0)	0.742
**MACE**	6 (9.1)	7 (30)	0.0131	3 (2.4)	2 (14.3)	0.0248

Data are as follows: median (25–75 percentile) for continuous variables or values (%) for categorical variables. Comparison made by # Mann-Whitney test for continuous variables and $ Chi square test for categorical variables. * *p* < 0.05. MACE: (death, non-fatal MI, unplanned revascularisation, stroke, and admission with angina); MTHR: Maximum Target Heart Rate; LV: left ventricle; SBP: systolic blood pressure; DBP: diastolic blood pressure; HR: heart rate; WMSI: wall motion score index; DSE: dobutamine stress echocardiography; BSA: Body Surface Area; CAD: coronary artery disease; ACE-I/ARB: angiotensin convertase enzyme inhibitor/angiotensin receptor antagonist. Double product (SBP*HR at peak). DHP: dihydropyridine.

Eighteen patients developed MACE over the 12-month follow-up period. There were five deaths, three non-fatal myocardial infarctions, two unplanned revascularisations, four strokes, and four hospital admissions with worsening angina (Table 3). The composite MACE was significantly higher in the group with-RCMx compared with the without-RCMx group (15% vs. 4%, *p* = 0.0028). A detailed summary of the MACEs is listed in Table 2. The main difference in the MACE was related to a higher number of non-fatal myocardial infarctions, unplanned revascularisations, and admissions with progressive angina amongst patients with RCMx during Dobutamine SE. There were equal stroke events between the groups and one extra death in the group without RCMx (Table 3).

### 3.5. Predictors of the Outcome Measures (THR, Dobutamine SE Outcome, and MACE)

**Figure 1:**
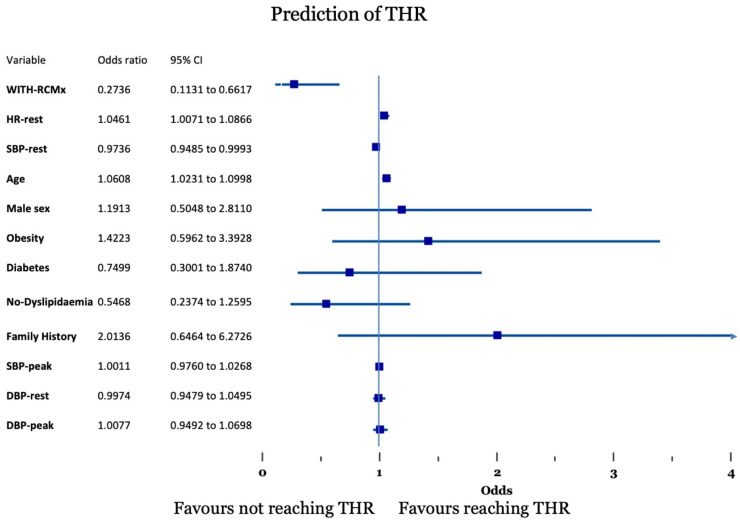
Forest plot of the predictors of target heart rate (THR).

The logistic regression and stepwise regression analysis showed that older age (OR 1.0608 1.0231–1.0998 and 1.059 1.023–1.096, respectively), higher resting HR (OR 1.0461 1.0071–1.0866 and 1.06 1.022–1.10, respectively), lower SBP at rest (OR 0.9736 0.9485–0.993 and 0.9756 0.958–0.993, respectively), and the lack of RCMx (OR 0.2736 0.1131–0.6617 and 0.311 0.140–0.867, respectively) were predicting whether patients achieved THR during Dobutamine SE (Figure 1, Supplementary Table S2).

**Figure 2:**
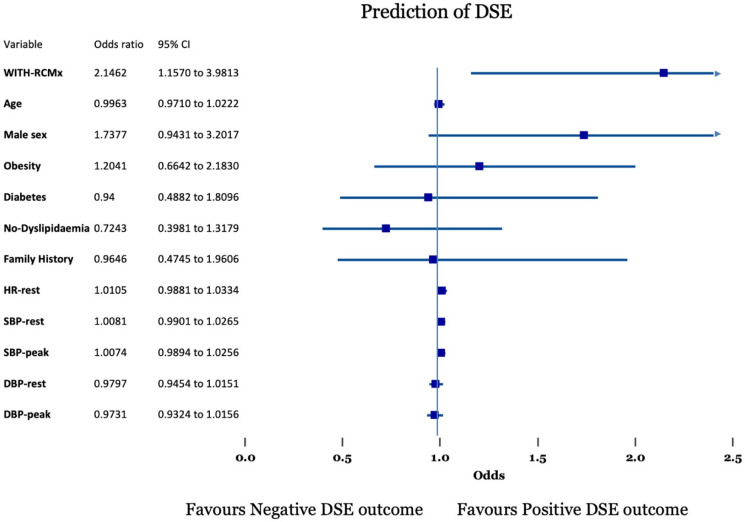
Forest plot of the predictors of Dobutamine stress echocardiography (DSE) outcome.

Both logistic regression and stepwise regression analysis confirmed that the only predictor of Dobutamine SE outcome was the usage of RCMx (OR 2.1462 1.1575–3.9813 and OR: 2.37 1.359–4.1414, respectively) (Figure 2, Supplementary Table S3).

**Figure 3:**
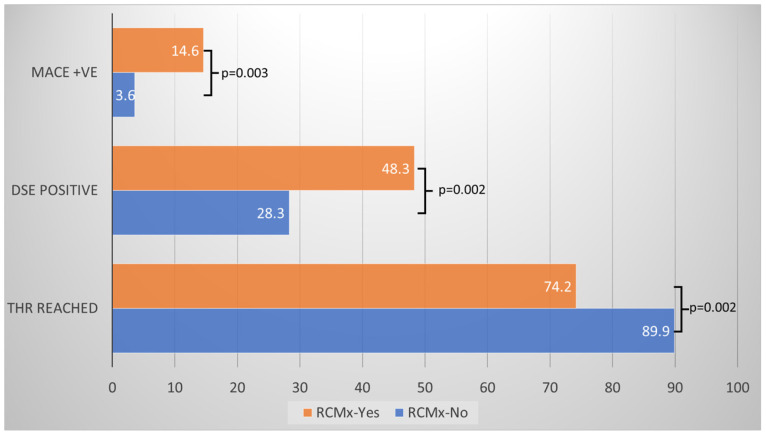
Main outcome of measurements in a function of the 2 groups: patients with (orange) and without (blue) RCMx. The bars represent the proportion of events in the two groups with and without RCMx.

The independent predictors of MACE using the stepwise regression model were RCMx (OR 3.6817 1.2271–11.0461) and WMSI at peak (as a sign of the extent of inducible ischaemia during Dobutamine SE) (OR 5.4371 1.1646–25.3830). The model rejected other confounding variables such as age, diabetes mellitus, dyslipidaemia, male sex, obesity, family history, SBP, DBP, and HR at rest and peak stress test, respectively (Figure 3. Supplementary Table S4).

## 4. Discussion

In this study, we found that the diagnostic efficacy of Dobutamine SE was not affected by the continuation of RCMx compared to those who were not on RCMx during the test. As expected, the THR was achieved in more patients without RCMx; however, the proportion of inducible ischaemia during Dobutamine SE was higher in patients with RCMx irrespective of whether they achieved THR or not (48% vs. 27% and 48% vs. 36%, respectively, Table 2).

In addition, MACEs were higher in patients who did not achieve THR in both the without-RCMx and with-RCMx groups (30% vs. 9%, and 14% vs. 2.4%, respectively). This observation is in keeping with the published data showing THR, during any form of stress echocardiography, as a predictor of all-cause mortality [5]. They also showed no effect of beta blocker treatment prior to SE in the prediction of outcomes [5]. Indeed, both the European echo database and the UK- EVEREST study reported that patients undergoing Dobutamine SE who had not achieved their THR (i.e., had chronotropic incompetence) showed a worse 5-year all-cause death rate and combined MACEs of cardiovascular death and non-fatal myocardial infarction [5,6]. We show that the two independent predictors of MACEs were RCMx and WMSI at peak stress. It is noteworthy that the traditional cardiovascular risk factors did not predict either Dobutamine SE outcome or MACEs.

### 4.1. Explanation of the Findings

One possible reason why patients with RCMx had more often positive Dobutamine SE despite the higher rate of chronotropic incompetency is that these patients had already been treated with beta blockers or rate-controlling CCB for angina with a higher likelihood of significant CAD. As the Dobutamine SE protocol, unless RWMA developed, the stress test continued with the addition of muscarinic receptor antagonists (atropine) and the initiation of a hand grip exercise, with the aim of achieving the THR, which may have resulted in a more extensive chronotropic and inotropic stimulation and thus increased myocardial oxygen consumption, which triggered inducible ischaemia even at a lower THR. Equally, the reverse hypothesis would be that patients without RCMx can achieve THR at an earlier stage of the stress test that would sometimes meet the criteria to terminate the test without allowing myocardial ischaemia to develop or persist due to the short time duration of the mismatch between oxygen supply and demand precluding the manifestation of the signs of inducible ischaemia. This hypothesis is supported by the frequently observed changes in LV wall segments kinesis/wall thickening properties in the early recovery phase [12]. It is important to note that myocardial perfusion imaging during SE can improve diagnostic accuracy, particularly in cases of chronotropic incompetence [13].

### 4.2. Clinical Translation

Our findings indicate that there should not be a pre-stress test recommendation to patients to omit their RCMx for the indication of chest pain assessment for suspected CAD. In fact, we observed more positive tests in patients with RCMx that translated to a higher incidence of MACEs. One may argue that omitting beta blockers—or other antianginal medication for a brief period prior to a stress test—may provoke rebound effects and could elicit unstable symptoms, even myocardial infarction [7,8,9]. The reduced sensitivity caused by RCMx has only been shown for patients who underwent a vasodilator stress test [7]. In current Western health care services, the flexibility to utilise the capacity for stress tests is important, and the lack of need for omitting RCMx 48 h prior the test allows for the more flexible scheduling of patients without disadvantaging patients.

## 5. Limitations

This study has several limitations. Firstly, the recruitment period was only a year prior the study; therefore, the number of patients was limited and, due to the short follow-up period of 12 months, the event rates were small, which in turn resulted in the wide confidence interval of the MACE resembling low precision. Nevertheless, the main question we wished to investigate was whether RCMx does effect Dobutamine SE outcomes. We did not perform a power calculation but targeted all consecutive patients referred for Dobutamine SE for chest pain assessment within the twelve months of the study period. Secondly, we only investigated stress tests performed with dobutamine as a stimulation agent. We cannot make comments regarding other forms of stress tests such as vasodilator SE or exercise-based stress tests. However, a vasodilator stress test’s main termination criteria is not the achieved age-predicted THR. On the other hand, our observation can be transferrable to exercise SE cases where THR is one of the termination criteria, especially if it is combined with a small dose of atropine administration at the peak. Thirdly, we had a few missing data, primarily the resting HR, SBP, and DBP, which needed median imputation for completing the analysis. This, however, did not affect either of the outcome measures’ predictions (Supplementary Table S1). Fourth, regarding the details of RCMx, 40% of the study population were prescribed RCMx, with 93% receiving β-blockers and 7% on rate-controlling calcium channel blockers; hence, the dominant effect on the outcome can be attributed to beta blocker treatment. Unfortunately, precise details regarding the specific preparations, dosages, and duration of therapy prior to SE were not available within this retrospective dataset.

Furthermore, although the initiation of new medications relative to SE referral cannot be confirmed, the average waiting time for SE in this cohort was approximately 3–4 months. Therefore, it is reasonable to assume that patients categorised as receiving RCMx would have been on these therapies for at least 3–4 months prior to their SE.

Finally, the variables included in the main analyses—age, sex, diabetes, obesity, dyslipidaemia, family history of coronary disease, and blood pressure—were selected based on established associations with cardiovascular outcomes and their relevance to the study population and the availability for all participants, ensuring consistency in adjustment. The severity of CAD and indication for RCMx were not uniformly documented and thus could not be reliably incorporated as covariates. Residual confounding remains a possibility, particularly given the observational design and limitations in available data. To mitigate this, we used stepwise regression to ensure only the most robust predictors were retained for the MACE outcome.

All continuous variables were assessed for normality and entered into the regression models as continuous predictors.

## 6. Conclusions

In this retrospective cohort study, we showed that the use of RCMx is a predictor of both SE and MACE outcomes irrespective of the achieved THR. Our data suggest that patients referred for Dobutamine SE whilst on RCMx can continue without impact on the test’s accuracy. This in turn augments the utilisation of health care resources by controlling and maximising diagnostic test capacity.

## Figures and Tables

**Table 1:** Patient characteristics, stress echocardiography, and MACE outcome.

	Total	WITH RCMx	WITHOUT RCMx	*p*
N	227	89 (39)	138 (61)	0.0012
Age	69 (59–76)	69 (61–77)	68 (55–75)	0.1191 #
Male sex	111 (49)	53(60)	58 (40)	0.010 $
Height (cm)	167 (160–174)	166 (160–176)	167 (160–173)	0.6600 #
Weight (kg)	80 (71–92)	82 (73–96)	77 (68–88)	0.0555 #
BSA (m^2^)	1.90 (1.70–2.00)	1.90 (1.80–2.10)	1.90 (1.70–2.00)	0.0879 #
**Cardiovascular risk factors**				
Hypertension	138 (61)	60 (67)	78 (57)	0.1015 $
Dyslipidaemia	117 (52)	49 (55)	68 (49)	0.3959 $
Smoker	44 (19)	16 (18)	28 (20)	0.3523 $
Former smoker	27 (12)	14 (16)	13 (9)	0.1105 $
Diabetes mellitus	66 (29)	37 (42)	29 (21)	0.0009 $
Obesity	81 (36)	38 (43)	43 (31)	0.0771 $
Family history of CAD	53 (23)	17 (19)	36 (26)	0.2255 $
**Medications**				
Beta receptor blockers	83 (37)	83 (93)	0 (0)	<0.0001 $
Rate-controlling calcium channel blockers	6 (3)	6 (7)	0 (0)	0.0020 $
DHP-calcium channel blockers	63 (28)	28 (31)	35 (25)	0.3175 $
ACE-I/ARB	98 (43)	52(58)	46 (33)	0.0002 $
Anti-platelets	116 (51)	60 (67)	56 (41)	0.0001 $
Nitrate	64 (28)	35 (39)	29 (21)	0.0028 $
Other antianginals	17 (8)	11 (12)	6 (4)	0.0255 $
**DSE parameters**				
HR at rest (bpm)	71 (63–80)	68 (57–78)	74 (64–81)	0.0004 #
HR at peak (bpm)	135 (127–144)	132 (122–140)	140 (130–147)	<0.0001 #
SBP rest (mmHg)	138 (120–160)	138 (119–159)	140 (120–160)	0.7490 #
SBP peak (mmHg)	136 (120–158)	137 (122–159)	135 (119–156)	0.5261 #
DBP rest (mmHg)	74 (67–80)	72 (65–78)	76 (68–82)	0.0219 #
DBP peak (mmHg)	67 (58–74)	65 (58–74)	67 (58–75)	0.4687 #
THR achieved	190 (84)	66 (74)	124 (90)	0.0018 $
THR at peak (% of MTHR)	88 (85–92)	87 (84–91)	89 (86–93)	0.0038 #
Double product (mmHg*bpm)	18,304 (16,037–21,714)	18,161 (15,358–21,223)	19,040 (16,256–22,032)	0.0852 #
WMSI at rest	1.00(1.00–1.06)	1.00 (1.00–1.06)	1.00 (1.00–1.00)	0.0702 #
WMSI at peak	1.00(1.00–1.12)	1.06 (1.00–1.20)	1.00 (1.00–1.06)	<0.0001 #
Delta WMSI	0.000 (0.000–0.060)	0.00 (0.00–0.19)	0.00 (0.00–0.060)	0.0002 #
Positive DSE (inducible ischaemia)	82 (36)	43(48)	39 (28)	0.0022 $
Biphasic response (viable ischaemic)	39 (18)	21 (24)	18 (13)	0.0329 $
**Number of inducible ischaemic LV segments**				
0	144 (63)	40 (43)	104 (77)	0.0001 $
1–2	60 (26)	38 (41)	22 (16)	0.0001 $
3–4	20 (9)	13 (14)	7 (5)	0.0180 $
≥5	3 (1)	1 (1)	2 (1)	1.000 $
**MACE**	18 (8)	13 (15)	5 (4)	0.0028 $

Data are as follows: median (25–75 percentile) for continuous variables or values (%) for categorical variables. Comparison made by # Mann–Whitney test for continuous variables and $ Chi square test for categorical variables. * *p* < 0.05. MACE: (death, non-fatal MI, unplanned revascularisation, stroke, and admission with angina); MTHR: Maximum Target Heart Rate; LV: left ventricle; SBP: systolic blood pressure; DBP: diastolic blood pressure; HR: heart rate; WMSI: wall motion score index; DSE: dobutamine stress echocardiography; BSA: Body Surface Area; CAD: coronary artery disease; ACE-I/ARB: angiotensin convertase enzyme inhibitor/angiotensin receptor antagonist. Double product (SBP*HR at peak). DHP: dihydropyridine.

**Table 3:** Components of the MACEs for the two groups with and without RCMx.

MACEs	With RCMx (n = 13)	Without RCMx (n = 5)
**Death**	**2**	**3**
**Non-fatal MI**	**3**	**0**
**Unplanned revascularisation**	**2**	**0**
**Stroke**	**2**	**2**
**Admission with progressive angina**	**4**	**0**

MACEs: major adverse cardiovascular events; RCMx: rate-controlling medical treatment; and MI: myocardial infarction.

## Data Availability

Data used in this study can be available from the corresponding author at reasonable request.

## Supplementary Materials

The following supporting information can be downloaded at: https://www.mdpi.com/article/10.3390/jcm15082850/s1, Table S1: Subgroup characteristics as a function of THR achieved in the two groups with or without RCMx both with and without median imputation for missing data handling (without missing data in red). Table S2: Determinants to predict achieved THR (logistic regression analysis). Table S3: Determinants to predict positive DSE outcome (logistic regression analysis). Table S4: Determinants of MACE (stepwise regression analysis).

## Abbreviations and Acronyms

RCMx	Rate Controlling Medical Treatment
WMSI	Wall Motion Score Index
CAD	Coronary Artery Disease
THR	Target Heart Rate
SE	Stress Echocardiography
MACE	Major Adverse Cardiovascular Event
MPS	Myocardial Perfusion Scintigraphy
SE	Stress Echocardiography
RCT	Randomised Control Trials
CCTA	Coronary Computed Tomography Angiography
ECG	Electrocardiogram
SPECT	Single-photon Emission Computed Tomography
ESC	European Society of Cardiology
