# Supplementary material for: The Effect of Rate-Controlling Medication on the Performance and Outcome of Dobutamine Stress Echocardiography in the Assessment of Patients with Suspected Angina: A Retrospective Cohort Study
Source: J Clin Med. 2026 Apr 9;15(8):2850. doi: 10.3390/jcm15082850 (PMC13116355; doi:10.3390/jcm15082850)
Supplement: Supplementary file 1 [file jcm-15-02850-s001.zip › jcm-4217865-supplementary.pdf]

SUPPLEMENTARY DOCUMENTS

The effect of rate controlling medication on the performance and outcome of dobutamine stress echocardiography in the assessment of patients with suspected angina
(A quality improvement project)

Laya Hariharan[1], Muhammad Amjad[1], Emil John[1], Valentina Cospite[2], Sudipta Chattopadhyay [1,3], Attila Kardos [1,4]

Affiliations

1. Department of Cardiology, Translational Cardiovascular Research Group, Milton Keynes University Hospital NHS Foundation Trust, Milton Keynes, United Kingdom

2. Department of Cardiology, County Durham and Darlington NHS Foundation Trust, Durham, United Kingdom

3. Department of Cardiology, Bedford Hospital NHS Trust, Bedford, United Kingdom

4. Faculty of Medicine and Health Sciences, University of Buckingham, Buckingham, United Kingdom

Correspondence

Prof. Attila Kardos, MD, FRCP, PhD, FESC,
Department of Cardiology, Translational Cardiovascular Research Group, Milton Keynes University Hospital NHS Foundation Trust, Milton Keynes, 8H Standing Way, Eaglestone, Milton Keynes, MK6 5LD United Kingdom.

Email: attila.kardos@cardiov.ox.ac.uk

Supplementary Table S1. Subgroup characteristics as a function of THR achieved in the two groups with or without RCMx both **with and without median imputation for missing data** handling (without missing data in red) (**Supplementary Table S1**)

	**WITH – RCMx (N=89)**			**WITHOUT – RCMx (N=138)**		
	**THR achieved**	**THR not achieved**	p	**THR achieved**	**THR not achieved**	p
N	66 (74%)	23 (26%)	<0.001	124 (90%)	14 (10%)	<0.001
Age	71 (61-77)	67 (60-73)	0.1118	69 (56-750	59 (51-66)	0.0515
Male Sex	40 (61)	13 (57)	0.7325	51(41)	7(50)	0.5254
Height (cm)	166 (160-174)	168 (160-181)	0.2202	166 (160-173)	170 (157-177)	0.5064
Weight (kg)	84 (73-97)	79 (65- 94)	0.2747	77 (67-87)	85 (80-111)	0.0127
BSA (m^2)	1.90 (1.80-2.10)	1.90 (1.72-2.18)	0.7775	1.90 (1.70-2.00)	2.00 (1.87-2.12)	0.0131
**Cardiovascular risk factors**						
Hypertension	46 (70)	14 (61)	0.4393	69(56)	9(64)	0.538
Dyslipidaemia	38(58)	11(48)	0.4209	64 (52)	4 (29)	0.1034
Smoker	11 (17)	5(22)	0.2154	27(22)	1(7)	0.3924
Diabetes Mellitus	32 (49)<br>26 (39%)	6 (48)<br>11 (48%)	0.4823<br>0.4823	27(22)	2 (14)	0.5160
Obesity	33 (47)<br>32 (48.5%)	6 (26)<br>6 (26%)	0.0629<br>0.0629	37(30)	6(43)	0.3205
Family History of CAD	13 (20)	4 (17)	0.8097	35(28)	1(7)	0.0897
**Medications**						
Beta receptor blockers	62(94)	21(91)	0.666	0 (0)	0(0)	1.000
Rate controlling Calcium Channel blockers	4(6)	2(9)	0.666	0(0)	0(0)	1.000
DHP-Calcium Channel blockers	22(33)	6(26)	0.5612	29 (23)	6 (43)	0.1138
ACE-I/ARB	38 (58)	14 (61)	0.7837	40(32)	6(43)	0.4269
Anti-platelets	44(67)	16(70)	0.799	50 (40)	6(43)	0.8553
Nitrate	28(42)	7(30)	0.3135	26(21)	3(21)	0.9681
**DSE parameters**						
HR at rest (bpm)	69 (60-79)	59 (56-69)	0.0232	74 (64-81)	72 (63-82)	0.5242
HR at rest (bpm) - imputed	69.5 (60.0-78.0)	63.0 (56.0-71.0)	0.051	73 (64-80)	72 (63-82)	0.5462
HR at peak (bpm)	135 (128-143)	116 (114-124)	<0.001	140 (130-148)	131 (126-136)	0.0139
SBP rest (mmHg)	136 (117-161)	147 (129-156)	0.2911	139 (120-159)	150 (124-175)	0.2251
SBP rest (mmHg)- imputed	137 (117-160)	138 (130.3-155.3)	0.305	138 (120-157)	150 (124-175)	0.2318
SBP peak (mmHg)	136 (122-154)	141 (123-169)	0.3906	135 (118-155)	135 (121-177)	0.5421
SBP peak (mmHg) - imputed	136 (122-153)	139 (124-169)	0.3484	136 (119-154)	135 (121-177)	0.5701
DBP rest (mmHg)	72 (64-78)	72 (67-78)	0.7645	75 (68-82)	78 (72-82)	0.2205

DBP rest (mmHg) - imputed	72 (64-77)	74 (67-78)	0.686	74 (68-82)	78 (72-82)	0.1965
DBP peak (mmHg)	65 (58-73)	65 (59-77)	0.4351	67 (59-74)	68 (58-78)	0.7161
DBP peak (mmHg) - imputed	67 (58-72)	67 (59-76)	0.502	67 (60-74)	68 (58-78)	0.7110
THR at peak (% of MTHR)	89 (87-92)	79 (71-81)	<0.001	89 (87-94)	84 (81-84)	<0.001
Double product (mmHg*bpm)	18681 (15799-21870)	16144 (14720-18810)	0.0445	18810 (16255-22028)	18176 (16214-23010)	0.9186
Double product (mmHg*bpm)	18617 (16320-21708)	16100 (14707-18697)	0.0189	19040 (16479-22024)	18176 (16214-3010)	0.8105
WMSI at rest	1.00 (1.00-1.06)	1.00 (1.00-1.04)	0.7714	1.00 (1.00-1.00)	1.00 (1.00-1.06)	0.2229
WMSI at peak	1.06 (1.00-1.18)	1.06 (1.00-1.23)	0.7896	1.00 (1.00-1.06)	1.00 (1.00-1.06)	0.7208
Delta WMSI	0.00 (0.00-0.18)	0.00 (0.00-0.18)	0.7222	0.00 (0.00-0.060)	0.00 (0.00-0.060)	0.4998
Positive DSE (inducible ischaemia)	32 (48)	11(48)	0.9568	34(27)	5(36	0.5150
Biphasic response (viable - ischaemic)	16(24)	5(22)	0.8858	14 (11)	4(29)	0.089
**Number of LV segments with inducible ischaemia**						
0	33(50)	12(52)	0.680	90(73)	9(64)	0.839
1-2	20 (30)	7 (30)	0.791	30(24.0)	3(21	0.486
3-4	12 (18)	3(13)	0.478	3(2)	2(14)	0.045
≥5	1 (2)	1 (4)	0.553	1(1)	0(0.0)	0.742
**MACE**	6(9.1)	7(30)	0.0131	3(2.4)	2(14.3)	0.0248

Data are: Median, (25-75 percentiles) for continuous variables, or values, (%) for categorical variables. Comparison made by #Mann-Whitney test for continuous variables and $Chi square test for categorical variables. *P<0.05. MACE: (death, non-fatal MI, unplanned revascularisation, stroke, admission with angina), MTHR: Maximum Target Heart Rate. LV: left ventricle. SBP: systolic blood pressure, DBP: diastolic blood pressure, HR: heart rate, WMSI: wall motion score index, DSE: dobutamine stress echocardiography, BSA: Body Surface Area. CAD: coronary artery disease. ACE-I/ARB: angiotensin convertase enzyme inhibitor/Angiotensin receptor antagonist. Double product (SBP*HR at peak). DHP: dihydropyridine.

The non-imputed data are highlighted red, the imputed data in blue

Supplementary Table S2.

Determinants to predict achieved THR (logistic regression analysis)

Variable	Odds ratio	95% CI	P
**Age**	1.0608	1.0231 to 1.0998	0.0014
Male	1.1913	0.5048 to 2.8110	0.6895
Obesity	1.4223	0.5962 to 3.3928	0.4271
Diabetes	0.7499	0.3001 to 1.8740	0.5379
No-Dyslipidaemia	0.5468	0.2374 to 1.2595	0.1562
Family Hx of CAD	2.0136	0.6464 to 6.2726	0.2273
**HR_at_rest_**	1.0461	1.0071 to 1.0866	0.0199
**On RCMx**	0.2736	0.1131 to 0.6617	0.004
**SBP_at_rest**	0.9736	0.9485 to 0.9993	0.0445
SBP_peak_	1.0011	0.9760 to 1.0268	0.9353
DBP_at_rest	0.9974	0.9479 to 1.0495	0.9207
DBP_peak_	1.0077	0.9492 to 1.0698	0.8012

Supplementary Table S3.

Determinants to predict positive DSE outcome (logistic regression analysis)

Variable	Odds ratio	95% CI	P
Age	0.9963	0.9710 to 1.0222	0.7769
Gender_M_1_F_0=1	1.7377	0.9431 to 3.2017	0.0764
Obesity_BMI_30_1_0=1	1.2041	0.6642 to 2.1830	0.5407
Diabetes_1_0=1	0.94	0.4882 to 1.8096	0.853
Dyslipidaemia_1_0=0	0.7243	0.3981 to 1.3179	0.2909
Family_Hx_1_0=1	0.9646	0.4745 to 1.9606	0.9206
HR_at_rest_	1.0105	0.9881 to 1.0334	0.3627
**ON RCMx**_1_0=1	2.1462	1.1570 to 3.9813	0.0154
SBP_at_rest_	1.0081	0.9901 to 1.0265	0.3811
SBP_peak_	1.0074	0.9894 to 1.0256	0.4223
DBP_at_rest_	0.9797	0.9454 to 1.0151	0.2577
DBP_peak_	0.9731	0.9324 to 1.0156	0.211

Supplementary Table S4.

Determinants of MACE (stepwise regression analysis)

Variable	Odds ratio	95% CI	P
RCMx_1_0=1	3.6817	1.2271 to 11.0461	0.0201
WMSI_peak	5.4371	1.1646 to 25.3830	0.0313

Variables not included in the model
Age
DBP_at_rest
DBP_peak
Diabetes_1_0=1
Dyslipidaemia_1_0=0
Gender_M_1_F_0=1
Obesity_BMI_30_1_0=1
Family_Hx_1_0=1
HR_at_peak
HR_at_rest_
SBP_at_rest_
SBP_peak
